# Rare Presentation of Heterozygous PCSK1 Deficiency in an Adolescent Male

**DOI:** 10.1155/crpe/3460735

**Published:** 2026-02-27

**Authors:** Tai Metzger, Abdullah Jalal, Silvestre R. Duran

**Affiliations:** ^1^ Department of Foundational Medical Studies, Oakland University William Beaumont School of Medicine, 586 Pioneer Dr, Rochester, 48309, Michigan, USA, oakland.edu; ^2^ Pediatric Cardiology, Corewell Health William Beaumont University Hospital, 3601 W 13 Mile Rd, Royal Oak, 48073, Michigan, USA

## Abstract

**Background:**

Proprotein convertase subtilisin/kexin type 1 (PCSK1) is an enzyme involved in processing prohormones into active peptides. PCSK1 deficiency is a rare genetic condition in which the homozygous presentation has been documented to cause diarrhea during infancy, as well as childhood obesity, high levels of proinsulin, and diverse endocrine abnormalities.

**Case Description:**

An eleven‐year‐old male was evaluated in the pediatric cardiology clinic for hypertriglyceridemia and rapid weight gain. He had recently been diagnosed with heterozygous PCSK1 deficiency, defined as c.661A > G, which is predicted to result in the amino acid substitution p.Asn221Asp. The patient reported regular hyperphagia to the point of nausea, with a diet of processed and sugary foods. Past medical history included obstructive sleep apnea and migraines. Physical examination was unremarkable aside from severe obesity (BMI 39.7 kg/m^2^) and elevated blood pressure. His fasting lipid panel showed elevated triglycerides (330 mg/dL), low HDL (38 mg/dL), normal LDL (71 mg/dL), elevated total cholesterol (175 mg/dL), and normal HbA1c (5.0%). The patient was counseled on lifestyle modifications with the weight management clinic and began a structured weight loss program along with discussions of possible GLP‐1 agonist initiation. Follow‐up lipid monitoring was planned in 3 months after the cardiology clinic visit.

**Discussion:**

This case of a heterozygous PCSK1 variant may demonstrate an association between this variant and the patient’s clinical presentation, possibly expanding the known clinical spectrum of the disorder beyond the previously reported presentations in homozygous cases. Our case may show how heterozygous presentations with this variant of PCSK1 deficiency demonstrate a different presentation from the homozygous phenotype in younger patients. This patient shows that PCSK1 abnormalities could have an association with individuals who have hyperphagia and significant obesity, but normal HbA1c and LDL levels. Additional studies could be considered to evaluate prevalence in the population, long‐term outcomes, and targeted therapies.

## 1. Introduction

Proprotein convertase subtilisin/kexin type 1 (PCSK1) is an enzyme involved in processing prohormones into active peptides. Deficiencies of this enzyme have been associated with decreases in multiple hormone levels, including insulin, thyroid hormones, cortisol, sex hormones, growth hormone, and hormones involved in satiety [1]. PCSK1 deficiency is a rare autosomal recessive condition, with about 30 reported cases worldwide [2]. Among the confirmed cases, the typical presentation of this disease includes persistent diarrhea during infancy, followed by polyuria and polydipsia in early childhood. Additionally, manifestations include early‐onset obesity and high levels of proinsulin, as well as other endocrine abnormalities [1, 3]. Treatment typically involves managing the patient’s obesity and replacing deficient hormones.

Here, we present a case of a PCSK1 variant in an 11‐year‐old male patient who had normal hemoglobin A1c levels and normal levels of low‐density lipoprotein (LDL). The patient did not have the infantile presentation but was rather found after referral to pediatric weight management and pediatric cardiology to evaluate his unusual lipid profile suspected to be caused by a potential PCSK1 deficiency.

## 2. Case Presentation

An eleven‐year‐old male presented to the pediatric cardiology clinic for evaluation of hypertriglyceridemia. His most recent fasting lipid panel obtained by his pediatrician showed elevated triglycerides (330 mg/dL), normal LDL (71 mg/dL), low HDL (38 mg/dL), elevated total cholesterol (175 mg/dL), and normal hemoglobin A1c (5.0%). Labs showed elevated fasted insulin (187 mlU/mL) and normal TSH (4.03 μIU/mL). His parents expressed concern about his rapid weight gain, noting that he eats to the point of nausea yet continues to feel hungry. His diet consisted mostly of processed and sugary foods as well as sugary drinks despite efforts to eat healthier.

The patient was previously found to have a heterozygous PCSK1 variant defined as c.661A > G, which, per the molecular genetics report through Prevention Genetics, is predicted to result in the amino acid substitution p.Asn221Asp. The report stated that this variant is a risk factor for obesity.

Past medical history included obesity (> 99th percentile), obstructive sleep apnea, and migraines. Family history was notable for hypothyroidism in the father and obesity in both parents. Physical examination was remarkable for elevated blood pressure (127/69), an elevated weight of 106 kg (> 99^th^ percentile), and a BMI of 39.7 kg/m^2^ (> 99^th^ percentile, Z‐score 2.7). Height was 1.6 m. The rest of the physical examination was unremarkable.

The patient was recommended to make dietary modifications to lower his triglycerides, including avoiding sugary foods and saturated fats, and to increase physical activity. Repeat fasting lipid panel to re‐evaluate triglycerides was scheduled for 3 months. The patient was also planned to follow up with the weight management clinic where initiation of GLP‐1 agonist would be discussed.

## 3. Discussion

Our case suggests a possible association between an atypical obesity phenotype and a heterozygous PCSK1 variant. With the patient’s degree of obesity in combination with his reported unhealthy diet, one would expect an elevated LDL level and possibly increased hemoglobin A1c. We sought to determine to what extent his presentation could be attributed to his genetic variant.

Both homozygous and heterozygous PCSK1 abnormalities have been shown to cause obesity. A 2016 review article by Stijnen et al. [4] demonstrated patients with homozygous or compound heterozygous loss‐of‐function mutations in PCSK1 leading to early‐onset obesity. These patients also had abnormal glucose homeostasis as well as malabsorptive diarrhea, hypogonadotropic hypogonadism, and multiple hormonal deficiencies. Creemers et al. identified eight rare variants that had an 8.7‐fold increased risk of obesity, suggesting that rare heterozygous PCSK1 mutations may contribute to the genetic predisposition for severe obesity in some individuals [5]. Other studies have shown that not all variants carry this association. Dijck et al. [6] investigated the association between the rare heterozygous PCSK1 p.Y181H variant and obesity in a Belgian population, finding no significant association with obesity. The authors suggest that while some rare heterozygous *PCSK1* variants—particularly protein‐truncating ones—may modestly influence BMI, most nonsynonymous variants like p.Y181H likely have limited clinical relevance for obesity [6]. Loffler et al. described variations in PCSK1 among obese children that resulted in altered glucose metabolism and endocrinopathies [7]. Similarly, Folon et al. [8] conducted a case–control study that included sequencing all coding exons of PCSK1 to assess the impact of rare heterozygous PCSK1 variants on obesity. Among 65 rare variants identified, only complete loss‐of‐function or null variants were significantly associated with obesity and increased BMI.

The effects of different PCSK1 variants on insulin regulation have previously been investigated and could explain the normal hemoglobin A1c seen in our patient. Stijnen et al. hypothesized that the increased proinsulin compared to insulin could contribute to the postprandial hypoglycemia seen in some PCSK1‐deficient patients as proinsulin has a longer half‐life than insulin, resulting in a net hypoglycemic effect [4]. A similar hypoglycemic effect was reported in the same paper in multiple summarized case reports of human patients. They describe postprandial hypoglycemia that could explain the normal hemoglobin A1c levels in obese patients. The role of PCSK1 regulation for appetite was further investigated in a mouse model from Martin et al. [9]. This study reduced the activity of prohormone convertase 1/3 (PC1/3). This prohormone is encoded by PCSK1 and plays a critical role in processing neuropeptides and hormones central to metabolic and endocrine regulation. Mice with reduced PC1/3 activity exhibited early‐onset obesity, hyperphagia, increased fat mass, and elevated serum leptin levels despite normal levels of insulin and corticosterone [9].

Some research suggests the potential of PCSK1 mutations to have a protective effect on hemoglobin A1c and LDL. One study in rodents with loss‐of‐function PCSK1 mutations found similar HDL cholesterol levels but increased LCAT and APOA1, enzymes involved in maintaining HDL levels [10]. The authors also found that while PCSK1 mutations are linked to obesity in humans, LDL levels decreased following a loss‐of‐function mutation [10]. Another study in humans found that a different PCSK1 variant may be associated with protection from the development of Type 2 diabetes despite being linked to increased body weight [11]. The authors hypothesized that this may be related to increased insulin levels, postprandial hypoglycemia, or increased GLP‐1 [11].

Our specific variant has not undergone in vitro enzymatic assays assessing variant function and therefore cannot, at this time, clearly demonstrate a cause for our patient’s presentation. Additional labs such as proinsulin, c‐peptide, HOMA‐IR, cortisol, and ACTH may support the association but are currently missing. Although this patient carries the heterozygous p.Asn221Asp (c.661A > G) PCSK1 variant, it is important to emphasize that this substitution should not be interpreted as a definitive causal mutation for an early‐onset PCSK1 deficiency syndrome. Instead, this variant is better understood as a potential susceptibility or modifier allele that may contribute to metabolic disturbances in combination with environmental influences, dietary patterns, and additional genetic factors.

## 4. Conclusion

This case overlaps with the early‐onset obesity described in previous case reports but differs in that our patient was heterozygous, had normal TSH, and was older than the other case reports. Thus, we expand the literature regarding PCSK1 variants beyond the previously described homozygous cases of diarrhea during infancy. Our case demonstrates a patient whose phenotype could, in part, be explained by a PCSK1 variant. Though the association cannot be proven at this time, the case underscores the importance of genetic testing in obese patients with atypical presentations. Though the short‐term management is unlikely to change for this patient, the long‐term management may change with advances in this field.

## Author Contributions

Tai Metzger: conceptualization, formal analysis, investigation, methodology, project administration, validation, visualization, writing–original draft, writing–review and editing, software; Abdullah Jalal: conceptualization, formal analysis, investigation, methodology, validation, writing–original draft, writing–review and editing, project administration; Silvestre R. Duran: conceptualization, formal analysis, funding acquisition, investigation, methodology, project administration, resources, supervision, validation, writing–original draft, writing–review and editing.

## Funding

No funding was received for this research.

## Consent

No written consent has been obtained from the patients as there are no patient identifiable data included in this case report.

## Conflicts of Interest

The authors declare no conflicts of interest.

## Data Availability

The data that support the findings of this study are available on request from the corresponding author. The data are not publicly available due to privacy or ethical restrictions.
